# Magnesium Can Protect against Vanadium-Induced Lipid Peroxidation in the Hepatic Tissue

**DOI:** 10.1155/2013/802734

**Published:** 2013-05-13

**Authors:** Agnieszka Ścibior, Dorota Gołębiowska, Irmina Niedźwiecka

**Affiliations:** ^1^Department of Zoology and Invertebrate Ecology, Laboratory of Physiology and Animal Biochemistry, The John Paul II Catholic University of Lublin, 102 Kraśnicka Avenue, 20-718 Lublin, Poland; ^2^Centre of Interdisciplinary Research, Laboratory of Oxidative Stress, The John Paul II Catholic University of Lublin, 102 Kraśnicka Avenue, 20-718 Lublin, Poland

## Abstract

The protective effect of magnesium as magnesium sulfate (MS) on sodium-metavanadate- (SMV-) induced lipid peroxidation (LPO) under *in vivo* and *in vitro* conditions was studied. The 18-week SMV intoxication (Group II, 0.125 V_end_/mL) enhanced spontaneous malondialdehyde (MDA) generation in rat liver, compared with the control (Group I) and MS-supplemented animals (Group III, 0.06 Mg_end_/mL). Coadministration of SMV with MS (Group IV, SMV-MS) caused a return of the MDA level to the control value range. The effect seems to result from the Mg_end_-independent action and its antagonistic interaction with V_end_. The *in vitro* treatment of liver supernatants (LS) obtained from all the tested animals groups with selected exogenous concentrations of Fe_exg_ or V_exg_ exhibited enhanced MDA production, compared with spontaneously formed MDA. It also showed Mg_exg_-stimulating effect on LPO (LS I, Group I) and revealed that the changes in the MDA generation in LS IV (Group IV) might have resulted from the synergistic interactions of V_end_ with Fe_exg_ and V_exg_ and from the antagonistic interactions of Mg_end_ with Fe_exg_ and V_exg_. The findings allow a suggestion that adequate Mg intake for a specific period in the conditions of SMV exposure may prevent V-induced LPO in the liver.

## 1. Introduction

Lipid peroxidation (LPO) is a well-known free-radical process defined as oxidative deterioration of lipids. It is used as an indicator of oxidative stress (OS), which occurs when the balance between the production of reactive oxygen species (ROS) and free radicals (FR) overrides the antioxidant capability of the cells or tissues [1–3]. It may be one of the possible mechanisms underlying oxidative cellular damage caused by ROS, and it can be implicated in the pathogenesis of a number of diseases [2, 4]. The products formed during LPO such as aldehydes, inter alia, and malondialdehyde (MDA) are well known to have deleterious effects. They can alter biological membrane organization and modify proteins and DNA. On the other hand, they can also modulate signal transduction pathways, induce adaptive response, as well as increase tolerance against forthcoming OS by upregulating defense capacity [5, 6].

Vanadium (V), which is a widely distributed element, has a wide range of industrial use. It interplays environmentally, occupationally, and biologically with human life [7]. Its toxicity depends, inter alia, on the route of administration, chemical form, and oxidation state, which determines the extensive biological effects of this element [8]. Due to its harmful health effects [9], our particular interest has been focused on searching factors which might prevent the deleterious action of V and attenuate its prooxidant activity. 

As a redox-active metal, V may modulate the cellular redox potential and be involved in oxidative injury mechanisms. In certain conditions, it may enhance the generation of oxygen-derived reactive species and stimulate LPO [10]. Its prooxidant properties have been revealed in *in vivo* and *in vitro* conditions both by us [11, 12] and by some other researchers [10, 13–21]. On the other hand, antioxidant action of V [22], its insulin-like effects [23], and anticarcinogenic activity [24–26] have also been reported. 

In turn, the relatively non-toxic and nonredox reactive magnesium (Mg) cannot participate in redox reactions that yield FR. It may effectively protect against FR and peroxidative damage. Its inhibitory effects on LPO have been demonstrated *in vitro* [27, 28] and *in vivo* in various animal models, including rats, [29–32] as well as in human studies [33, 34]. The limitation of LPO by this element has also been revealed under the conditions of cadmium and mercury exposure in a rat model [35, 35]. However, in some conditions Mg may stimulate LPO causing OS. Its ability to elevate LPO has been revealed by us [11, 12] and by some other investigators [27]. 

The antioxidant potential of Mg and its beneficial role in limiting LPO and the strong prooxidant potential of V and its well-known toxicological impact as well as insufficient information about the possible protective influence of Mg on V-induced LPO prompted us to perform an experiment in a rat model to explore the hypothesis whether an 18-week administration of Mg as magnesium sulfate (MgSO_4_, MS, 0.06 mg Mg/mL) in combination with sodium metavanadate (NaVO_3_, SMV) will be able to effectively limit V-stimulated LPO in the liver. This organ is one of the sites of V accumulation and plays a major role in the storage, secretion and production of many important substances as well as in maintenance of homeostasis and detoxification allowing the body to function and live. The influence of exogenous Mg, V and Fe on LPO in liver supernatants (LS) and the effects of interactions between them, recognition of which may help in elucidation of the cellular mechanisms of the response to combinations of metals, have also been examined. 

## 2. Material and Methods

### 2.1. Chemicals and Reagents

NaVO_3_ (SMV), (MgSO_4_, MS), iron sulfate (FeSO_4_), and thiobarbituric acid (TBA) were obtained from Sigma Chemicals (St. Louis, MO, USA). All the other chemicals and reagents used were of analytical grade. 

### 2.2. Experimental Design

The experiment was conducted on 40 adult outbred albino male Wistar rats with average initial body weight about 267 g, which, following an adaptation period of 7 days in a room in controlled conventional conditions, were randomly divided into 4 groups (10 rats per group). All the rats were individually housed in stainless steel cages (one rat per cage) when the experiment was started. Every day over a 18-week period, all the rats had unlimited access to the rodent laboratory chow (Labofeed B; Fodder and Concentrate Factory, Kcynia, Poland) in the shape of pellets of 12 mm diameter and they received to drink: Group I (untreated control)—deionized water; Group II (SMV)—a water solution of NaVO_3_ at a concentration of 0.125 mg V/mL; Group III (MS) a water solution of MgSO_4_ at a concentration of 0.06 mg Mg/mL; Group IV (SMV-MS)— a water solution of NaVO_3_ and MgSO_4_ at the same concentrations as in Group II for NaVO_3_ and in Group III for MgSO_4_. Food, fluids, and deionized water were offered *ad libitum*. Throughout the 18-weeks period, body weight was obtained weekly and at the time of slaughter. Animals' behavior was also observed.

The stock solutions of NaVO_3_ and MgSO_4_ were replaced by freshly prepared solutions every 2 days. The daily intake of water and the solutions of SMV, MS, and SMV-MS were measured with a measuring cylinder and the water and fluid intake was expressed as mL/rat/24 h. In turn, the daily intake of V and Mg in the SMV- or/and MS-administered animals was estimated on the basis of the 24 h consumption of the SMV, MS, and SMV-MS solutions and expressed as mg/kg b.wt./24 h. However, the food intake was calculated on the basis of the 24 h consumption of food by the rats from all the groups (the remainder of food together with additional spillage was weighed and subtracted from the whole food that the rats received to eat) and expressed as g/rat/24 h. The V and Mg concentrations in drinking water were selected on the basis of our previous experiments conducted in a rat model [11, 12, 36] and studies of other researchers [37, 38]. The concentration of V was chosen to reveal its prooxidant potential, which was meant to be attenuated by the administration of this element in combination with Mg. The concentration of Mg was chosen to be not too high since Mg (as MgSO_4_) has been reported to induce diarrhea [39, 40]. 

After 18 weeks, all the rats were sectioned between 8:00 and 11:00 am and livers, which were used to prepare LS for determination of the MDA level, and other organs were dissected, directly washed in ice-cold physiological saline solution (0.9% NaCl), and weighed. The biological material that was not used immediately was stored frozen at –20°C or –80°C in a deep-freezer HFU 486 basic (bought as part of the Project entitled “Building of the Centre of Interdisciplinary Research” realized within the frame of the Operating Programme “Development of Eastern Poland” 2007–2013, Priority I: Modern Economy, Action I.3. The Advancement of Innovation, cofinanced by the European Regional Development Fund) (Thermo Fisher Scientific, Germany) until further analysis. The experiment was conducted according to the experimental protocol approved by the 1st Local Ethical Committee for Animal Studies in Lublin, Poland. 

### 2.3. Analytical Procedure

LSs, in which the MDA level was determined using TBA, were obtained from 40 outbred 6.5-month-old albino male Wistar rats. More details concerning the preparation of LS for measurement of MDA and the methodology of determination of this LPO marker have been described by us previously [11]. LSs obtained from all the groups of rats: LS I (from Group I, Control), LS II (from Group II, SMV), LS III (from Group III, MS), and LS IV (from Group IV, SMV-MS) were divided into a few parts and subsequently incubated (a) without an inductor: LPO spontaneous (LPO_spont._), (b) with 30 *μ*M FeSO_4_ (Fe_exg  30 *μ*M_), (c) with 100, 200, or 400 *μ*M SMV (V_exg  100,  200,  400 *μ*M_), or (d) with 100, 200, or 400 *μ*M MS (Mg_exg  100, 200,  400 *μ*M_). The MDA formed was calculated using the molar extinction coefficient 1.56 × 10^5^ M^−1^ cm^−1^ and the results were expressed in nmoles per gram of wet tissue (nmol/g wet tissue).

### 2.4. Statistical Analysis

The results were processed with the Statistica and SPSS, version 9.0 and 14.0 PL for Windows, respectively. The distribution patterns in the data were evaluated using the Shapiro-Wilk's normality test. The homogeneity of variances was verified employing Levene's test and sometimes also Hartley's Fmax, Cochran's C and Bartlett's tests. The two-way analysis of variance (2-way ANOVA) with the vanadium (V_end_) and magnesium (Mg_end_) factors and the *F* test were employed to indicate the significant effects of V_end_, Mg_end_, or the V_end_  × Mg_end_ interaction. In addition, the three-way ANOVA analysis of variance (3-way ANOVA) with exogenous iron (Fe_exg  30 *μ*M_), exogenous vanadium (V_exg  100,  200,  400 *μ*M_), and exogenous magnesium (Mg_exg  100,  200,  400 *μ*M_) factors as well as the *F* test were also employed to reveal significant effects of Fe_exg  30 *μ*M_, V_exg  100,  200,  400 *μ*M  _, or Mg_exg  100,  200,  400 *μ*M_. *F* values which had *P* values smaller than 0.05 were considered statistically significant. If the 2- or 3-way ANOVA tests demonstrated interactive effects between the elements used or trends toward those effects, subsequent calculations were done in order to describe the character of the interactions revealed (antagonistic or synergistic) [41]. The *post hoc* comparisons between the four individual groups were performed using Tukey's or T3 Dunnett's tests. Comparisons between spontaneous LPO and LPO modified exogenously by Fe_exg_, V_exg_ and Mg_exg_ were assessed by the *t*-test or Wilcoxon test for dependent samples. The Student's “*t*”-test for independent samples was also applied for the detection of significant differences in the consumed V doses between the rats in Groups II and IV and Mg doses between the rats in Groups III and IV. The differences were considered significant if the *P* values were smaller than 0.05. All the results are expressed as mean ± SEM.

## 3. Results

### 3.1. General Observation

No distinct differences in the physical appearance and motor behavior were observed during the 18 experimental weeks in most of the rats receiving the SMV or/and MS solutions to drink, compared with the control. Some of the rats which drank the SMV and MS solutions separately (Groups II and III, resp.) and in combination (Group IV) had gastrointestinal disturbances, which were probably caused by the ingestion of V or/and Mg. Only one rat from Group IV had one-day diarrhea in the third and eight week of the experiment. In turn, loose stool was observed in one rat in Group II and in three rats in Groups III and IV in the first or/and second week of the experiment. However, in two rats in Group II and in one in Group III loose stool was observed at the turn of fifth and sixth week of the experiment and at the turn of second and fifth week of the study, respectively.

### 3.2. Basic Parameters

**Figure 1:**
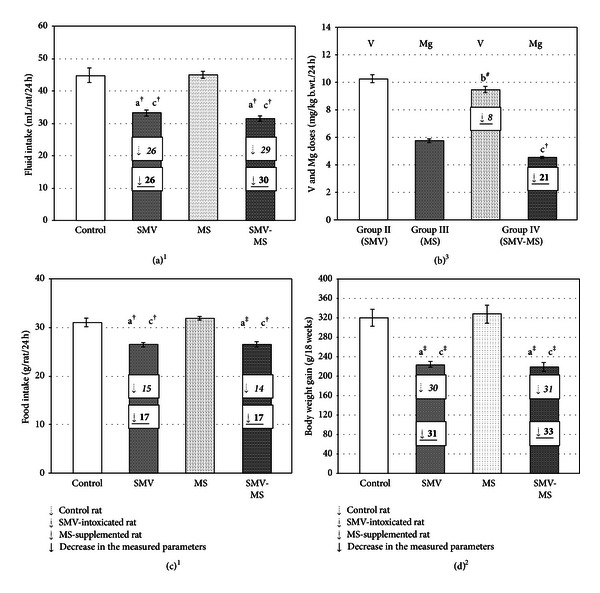
Fluid intake (a), V and Mg doses consumed by the rats through drinking water (b), food intake (c) and body weight gain (d) in the tested animals groups. Differences are indicated by ^a,b,c^versus control, SMV-intoxicated and MS-supplemented rats, respectively (^1,2,3^Tukey's, T3 Dunnett's and *t* test, resp.). *P < 0.05, ^‡^ *P* < 0.01, ^†^ *P* < 0.001, ^#^ *P* = 0.09. Numerical values in the bars indicate the percentage of the decrease in the measured parameters (↓), compared with the control (italic alone), the SMV-intoxicated (italic underline bold), and the MS-supplemented (underline bold) rats.

The fluid and food intakes as well as body weight gain in the rats of Groups II and IV were lower, compared with those found in the animals in Groups I and III (Figures 1(a), 1(c), and 1(d)). As the two-way ANOVA revealed, the decrease in the abovementioned parameters observed in the rats of Group IV was due to the independent action of V only (Table 1). It was also observed that the rats in Group IV took up slightly less V (by 8%), in comparison with the animals in Group II, but this difference did not turn out to be statistically significant. In turn, the consumption of Mg by the rats in Group IV was significantly lowered (by 21%), compared with that found in the animals in Group III (Figure 1(b)), which might be an effect of reduced fluid intake due to the SMV administration (Figure 1(a)).

### 3.3. Spontaneously Formed Hepatic MDA

**Figure 2:**
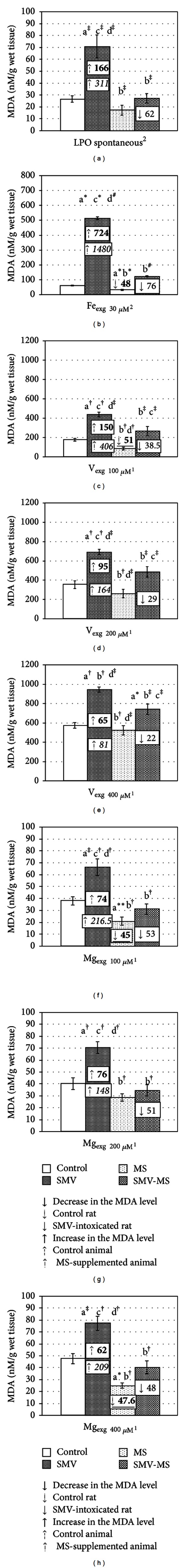
MDA level in LS obtained from the control, SMV-, MS- and SMV-MS-administered rats incubated without an oxidation inductor (LPO spontaneous) (a) or with Fe_exg  30 *μ*M  _ (FeSO_4_) (b), V_exg  100,  200,  400 *μ*M  _ (NaVO_3_) (c, d, e), or Mg_exg  100,  200,  400 *μ*M_ (MgSO_4_) (f, g, h). Differences are indicated by ^a,b,c,d^versus control, SMV-intoxicated, MS-supplemented, and SMV-MS-administered rats, respectively (^1^Tukey's and ^2^T3 Dunnett's test). *P < 0.05, ^‡^ *P* < 0.01, ^†^ *P* < 0.001, **P = 0.07, ^#^ *P* = 0.09, ^##^ *P* = 0.13. Numerical values in the bars or above them indicate the percentage of the decrease in the MDA level (↓), compared with the control (underline bold) and the SMV-intoxicated (normal alone) rats; other numerical values in the bars indicate the percentage of the increase in the MDA level (↑), compared with the control (bold alone) and the MS-supplemented (bold italic) animals.

As presented in Figure 2(a), the exposure to SMV alone (Group II) significantly enhanced the level of spontaneously generated MDA, compared with the control (Group I), the MS-supplemented (Group III) and the SMV-MS-applicated (Group IV) rats. Supplementation of the rats with MS alone did not change markedly the MDA formation, compared with the control, whereas the administration of MS in combination with SMV reduced its level by 62%, compared with the SMV-intoxicated rats. It was also observed that the level of the examined LPO marker was within the same value range that was found in the control animals. The two-way ANOVA revealed that the decrease in the spontaneously formed MDA in the rats of Group IV was influenced by the independent action of Mg and by its interaction with V (Table 1). 

### 3.4. MDA Level Modified by Fe_exg_, V_exg_, and Mg_exg_

In LS II, the MDA level modified by Fe_exg  30 *μ*M_ (Figure 2(b)), V_exg  100,  200,  400 *μ*M_ (Figures 2(c), 2(d), and 2(e)) or Mg_exg  100,  200, 400 *μ*M_ (Figures 2(f), 2(g), and 2(h)) increased markedly, compared with that found in LS I, III, and IV incubated in the same *in vitro* conditions. Further, in LS IV incubated with the concentrations of Fe_exg  30 *μ*M_, V_exg  100,  200,  400 *μ*M_, or Mg_exg  100,   200,  400 *μ*M_, the level of MDA was markedly decreased by 76%, 38.5%, 29%, 22%, 53%, 51%, and 48%, respectively, in comparison with that found in LS II incubated in the same manner (Figures 2(b)–2(h)). Moreover, in LS IV incubated with Fe_exg  30 *μ*M_ (Figure 2(b)) or with Mg_exg  100,  200,  400 *μ*M_ (Figures 2(f)–2(h)), the MDA level returned to the range of values obtained for LS I incubated with the same concentrations of Fe_exg_ and Mg_exg_. In the presence of V_exg  100 *μ*M_ or V_exg  200 *μ*M_ (Figures 2(c) and 2(d)), the level of this LPO marker was not significantly elevated, compared with that demonstrated in LS I. Only in the presence of the highest V concentration (V_exg  400 *μ*M_), its level was significantly higher, compared with LS I (Figure 2(e)). Furthermore, in LS IV incubated with V_exg  100,  200,  400 *μ*M_ (Figures 2(c), 2(d) and 2(e)), the MDA level was also significantly higher, compared with that found in LS III incubated in the presence of the abovementioned V_exg_ concentrations.

It was also shown that in LS III incubated in the presence of Fe_exg  30 *μ*M_ (Figure 2(b)), V_exg  100 *μ*M_ (Figure 2(c)), or Mg_exg  100,  400 *μ*M_ (Figures 2(f) and 2(h)), the level of MDA was lowered by 48%, 51%, 45%, and 47.6%, respectively, in comparison with that in LS I incubated with the same concentrations of Fe_exg_, V_exg_, or Mg_exg_. The level of MDA in LS III incubated with V_exg  200 *μ*M_, V_exg  400 *μ*M_ (Figures 2(d) and 2(e)), or Mg_exg  200 *μ*M_ (Figure 2(g)) was also lower, compared with that observed in LS I, but these differences were not so clear. 

**Figure 3:**
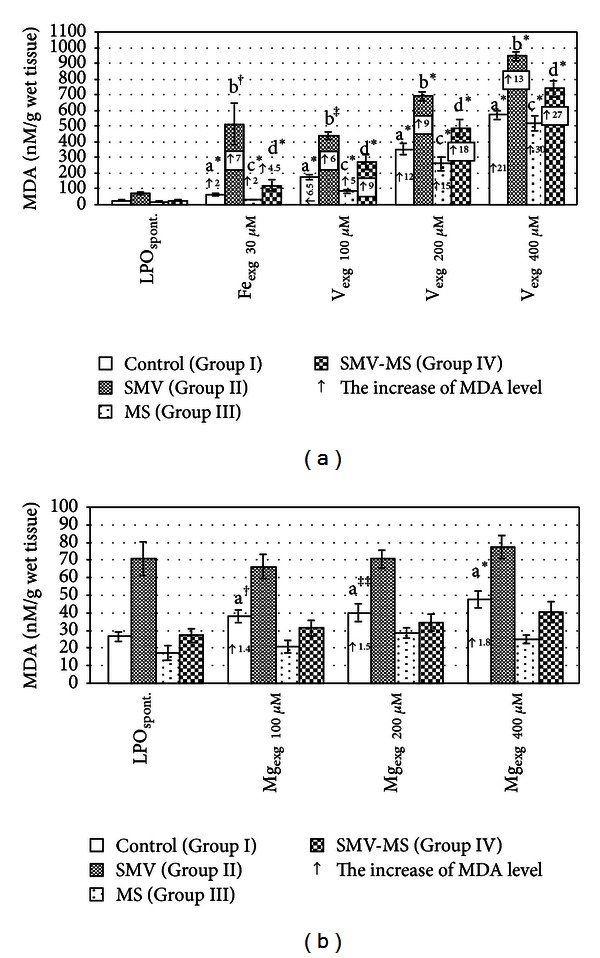
MDA level in LS obtained from the control, SMV-, MS- and SMV-MS-administered rats incubated without an oxidation inductor (LPO spontaneous) or with different exogenously added concentrations of Fe_exg  30 *μ*M_ or V_exg  100,  200,  400 *μ*M_ (a) or Mg_exg  100,  200,  400 *μ*M_ (b) as FeSO_4_, NaVO_3_ and MgSO_4_, respectively. Differences are indicated by ^a,b,c,d^versus Control_LPO spont._, SMV_LPO  spont._, MS_LPO  spont._ and SMV-MS_LPO  spont._, respectively, (Wilcoxon or *t* test). *P < 0.05, ^‡^ *P* < 0.01, ^†^ *P* < 0.001, ^‡‡^ *P* = 0.07. Numerical values in the bars or above them indicate how many times the MDA level increased (↑).

In addition, LS I, obtained from the control rats, which were incubated with Fe_exg  30 *μ*M_, V_exg  100,  200,  400 *μ*M_ or Mg_exg  100,  200,   400 *μ*M_ exhibited higher MDA production, compared with that observed in LS I incubated without (Control_LPO  spont._) the abovementioned concentrations of Fe_exg_ or V_exg_ (Figure 3(a)) or Mg_exg_ (Figure 3(b)). Higher MDA production was also demonstrated in LS II, III, and IV obtained from the SMV-intoxicated, MS-supplemented, and SMV-MS-administered rats, respectively, incubated in the presence of Fe_exg  30 *μ*M_ or V_exg  100, 200,  400 *μ*M_, in comparison with the spontaneously formed MDA in those LSs (Figure 3(a)). In turn, the incubation of LS II, III, and IV in the presence of Mg_exg  100,  200,  400 *μ*M_ did not significantly change the level of MDA, compared with SMV_LPO  spont._, MS_LPO  spont._, and SMV-MS_LPO  spont._, respectively (Figure 3(b)). 

The three-way analysis of variance revealed that the changes in the MDA level in LS IV (obtained from the rats supplemented with MS during the SMV exposure) modified by exogenous Fe_exg  30 *μ*M_, V_exg  100,  200,  400 *μ*M_, or Mg_exg  100,  200,  400 *μ*M_ resulted from the independent action of V_end_ and Mg_end_ as well as from their interaction or a distinct trend toward the V_end_  × Mg_end_ interaction (Table 2). In addition, the three-way ANOVA indicated that, beside the effects revealed between V_end_ and Mg_end_, the alterations in the MDA production in LS IV incubated with Fe_exg  30 *μ*M_ were also a consequence of the independent action of Fe_exg_ and its interaction with V_end_ and Mg_end_ as well as an effect of the interaction between three elements: Fe_exg_, V_end_, and Mg_end_. In turn, the changes in the MDA generation in LS IV incubated with V_exg  100  *μ*M_, V_exg  200  *μ*M_, or V_exg  400 *μ*M_ additionally resulted from the independent action of V_exg  100  *μ*M_, V_exg  200 *μ*M_, and V_exg  400 *μ*M_ and from their interaction with V_end_ and Mg_end_ (Table 2). In the case of incubation of LS IV with Mg_exg  200 *μ*M_ or Mg_exg  400 *μ*M_, the three-way analysis of variance revealed that the alterations in the level of MDA in LS IV in the presence of Mg_exg  200 *μ*M_ or Mg_exg  400 *μ*M_ were also an effect of independent action of Mg_exg  200 *μ*M_ and Mg_exg  400 *μ*M_. In turn, any significant effect of Mg_exg  100 *μ*M_ on the MDA production in LS IV was revealed by the performed analysis (Table 2).

## 4. Discussion 

The current report demonstrates the influence of the 18-week V and Mg administration (as SMV and MS, resp.), separately and in combination, on changes in such basic parameters as fluid and food intakes, and body weight gain in male Wistar rats. It also presents (a) the protective impact of Mg on the *in vivo* SMV-stimulated LPO in the rat liver, (b) the modulating effects of the exogenously used Mg, V, and Fe on LPO in *in vitro* conditions, (c) the main and interactive effects of the abovementioned elements, and (d) the character of their interactions with respect to changes in the explored free radical process. 

On the basis of the data obtained, we may state that the supplementation of the rats with MS during the 18-week SMV exposure did not limit the decrease in the fluid and food intake and body weight gain (Figures 1(a), 1(c) and 1(d)). Similar effects had also been observed by us previously in rats supplemented with MS during the 12-week SMV exposure [36, 42]. The changes in the fluid and food intake and in the body weight gain in rats after SMV intoxication had already been discussed [43]. 

As we expected, V (as SMV) enhanced LPO (Figures 1(a) and 3(a)). The elevated level of LPO in the liver of rats after intoxication with SMV or ammonium metavanadate (AMV) and in LS incubated with sodium vanadate in the *in vitro* system was also reported by other investigators [44–46]. A strong correlation between the induction of LPO and hepatotoxicity and the inhibition of both processes in parallel by antioxidants, suggesting a causative role for LPO in V-induced hepatotoxicity, was observed as well [47]. 

The performed analysis allowed us to conclude that the increase in the MDA production observed in LS IV (Group IV) in the presence of Fe_exg  30 *μ*M_ or V_exg  100,  200,  400 *μ*M_ (Figure 3(a)) was not only a consequence of the independent action of both elements but it also resulted from the synergistic interactions between Fe_exg_ and V_end_ and between V_end_ and V_exg_ (Table 2). The same interactive effects were found by us previously [11]. This is not surprising, as both elements may intensify LPO [48].

In turn, the incubation of LS II, III, and IV with Mg_exg  100,  200, 400 *μ*M_ did not significantly alter the MDA level, compared with spontaneously generated MDA, and only in LS I was a stimulating action of Mg on the hepatic MDA formation demonstrated (Figure 3(b)). The stimulating effect of Mg on the hepatic MDA production was also observed by us previously [11]. 

On the other hand, the present findings clearly demonstrated that the male Wistar rats receiving SMV in combination with MS (Group IV) for 18 weeks had a significantly lowered spontaneous MDA level than those exposed to SMV (Group II), in which the hepatic spontaneous MDA generation was markedly higher, compared with that found in the control (Group I) and MS-supplemented animals (Group III) (Figure 2(a)). The results obtained from the two-way ANOVA analysis allowed us to conclude that the protective impact of Mg on reduction of the SMV-stimulated hepatic MDA generation during the 18-week combined SMV and MS administration resulted from the independent action of Mg_end_ and from its antagonistic interaction with V_end_ (Table 1). Unfortunately, when the rats were supplemented with MS during the shorter 12-week SMV exposure, we did not demonstrate any significant fall in the spontaneously generated MDA in the liver, compared with that found in SMV-intoxicated rats [11]. We may suppose that the differences in the duration of the experimental period might be, at least partly, the cause of the discrepancies observed. 

In addition, the results of the three-way ANOVA analysis also allowed us to state that the limitation in the increase in the MDA production in LS IV incubated with Fe_exg  30 *μ*M_ or V_exg  100,  200, 400 *μ*M_, compared with LS II (Figures 2(b)–2(e)), might be associated with the antagonistic interaction of Mg_end_ with Fe_exg_ and V_exg_ (Table 2). Neither the antagonistic V_end_ × Mg_end_, Fe_exg_  × Mg_end_, and Mg_end_  × V_exg_ interactions nor the three-way interaction (Fe × V × Mg) (Table 2) had been observed by us previously [11]. 

An important new finding of the study is that the independent action of Mg_end_ was a major effect responsible for suppression of the spontaneously formed MDA in the liver of rats supplemented with MS during the SMV exposure (Table 1). We cannot exclude that the antiradical activity of Mg might underlie, at least in part, its beneficial effect [28, 49–51]. The effect of Mg on some antioxidants appears also worthy of inquiry [31, 52]. Therefore, further work is necessary to explain precisely the mechanism(s) responsible for the beneficial action of Mg in the 18-week conditions of the SMV-MS coadministration.

The V_end_  × Mg_end_ antagonistic interaction also played a significant role in the reduction of the SMV-induced spontaneous LPO in the liver of the SMV-MS-coadministered rats (Table 1). The V × Mg interactions investigated in *in vivo* and *in vitro* conditions are still little known, and only single reports about this issue have appeared in the literature [38, 53, 54]. Recently Sánchez et al. [55] showed that the interactions between V and Mg might occur in the rats' digestive and renal systems. The antagonistic character of the interaction revealed between V_end_ and Mg_end_ in our experimental conditions requires additional analyses. This seems to be important especially for extending the knowledge of the mechanism of the vanadate effect on organisms and the potential role of Mg in prevention of V toxicity. 

## 5. Conclusion

To the best of our knowledge, the current report is the first demonstration of the protective action of Mg against the prooxidant potential of V revealed in a rat model. The study has clearly demonstrated that the 18-week supplementation of male Wistar rats with Mg (as MS) during the exposure to V (as SMV) may protect against V-induced hepatic LPO. The study provides evidence that the beneficial influence of Mg on limitation of the increase in the hepatic MDA generation during the 18-week SMV intoxication may result from the independent action of Mg and from its antagonistic interaction with V. However, further studies are needed to explain the exact mechanism(s) accounting for the protective effect of Mg against the SMV-induced OS in our experimental conditions. The results obtained seem to suggest that a proper Mg intake for a specific time period in the conditions of SMV exposure may prevent V-stimulated LPO in the liver.

The present study has also shown the degree to which the independent action of the elements used (V, Mg, and Fe) and their mutual interactions may modify the hepatic MDA production. Simultaneously, it has confirmed that Mg is able to promote LPO in certain conditions by revealing its stimulating action on the explored free radical process in the *in vitro* system. 

## Figures and Tables

**Table 1:** Main and interactive effects of V_end_ and Mg_end_ on the measured variables in male Wistar rats after 18-week administration of both elements as SMV and MS in combination.

Variables^a^	Two-way ANOVA analysis^b^			Character of interaction revealed^c^
	Main effect of V_end_	Main effect of Mg_end_	Interactive effect of V_end_ × Mg_end_	
Fluid I	*F* = 61.263, *P* = 0.000	NS	NS	—
Food I	*F* = 45.645, *P* = 0.000	NS	NS	—
BWG	*F* = 46.591, *P* = 0.000	NS	NS	—
LPO_spontaneous_	*F* = 22.678, *P* = 0.000	*F* = 21.722, *P* = 0.000	*F* = 9.091, *P* = 0.005	Antagonistic

^a^Fluid I and Food I: fluid and food intake expressed as mL and g/rat/24 h, respectively; BWG: body weight gain expressed as g/18 week.

^b^Data are presented as *F* values and the levels of significance (*P*). NS: no significant effect.

^c^The effect of V_end_ and Mg_end_ in combination (V_end_ + Mg_end_ effect) < or > sum of the effects of V_end_ and Mg_end_ alone (V_end_ effect + Mg_end_ effect) (antagonistic or synergistic interaction, resp.).

**Table 2:** Main and interactive effects of V_end_, Mg_end_, Fe_exg_, V_exg_ and Mg_exg_ on the MDA level measured in liver supernatants obtained from the SMV-MS coapplied rats incubated in *in vitro* conditions with Fe_exg 30 *μ*M_, V_exg 100, 200, 400 *μ*M_ or Mg_exg 100, 200, 400 *μ*M_.

Three-way ANOVA analysis^a^		Character of interaction revealed or character of a trend toward interaction
LPO modified by Fe_exg 30 *μ*M_		
Main effect of V_end_	*F* = 18.412, *P* = 0.000	—
Main effect of Mg_end_	*F* = 11.626, *P* = 0.001	—
Interactive effect of V_end_ × Mg_end_	*F* = 8.097, *P* = 0.006	Antagonistic^b^
Main effect of Fe_exg 30 *μ*M_	*F* = 17.994, *P* = 0.000	—
Interactive effect of Fe_exg 30 *μ*M_ × V_end_	*F* = 12.317, *P* = 0.001	Synergistic^c^
Interactive effect of Fe_exg 30 *μ*M_ × Mg_end_	*F* = 6.995, *P* = 0.010	Antagonistic^d^
Interactive effect of Fe_exg 30 *μ*M_ × V_end_ × Mg_end_	*F* = 5.526, *P* = 0.021	

LPO modified by V_exg 100 *μ*M_		
Main effect of V_end_	*F* = 75.402, *P* = 0.000	—
Main effect of Mg_end_	*F* = 29.066, *P* = 0.000	—
Interactive effect of V_end_ × Mg_end_	*F* = 6.950, *P* = 0.051	Antagonistic^b^
Main effect of V_exg 100 *μ*M_	*F* = 206.252, *P* = 0.000	—
Interactive effect of V_exg 100 *μ*M_ × V_end_	*F* = 46.205, *P* = 0.000	Synergistic^e^
Interactive effect of V_exg 100 *μ*M_ × Mg_end_	*F* = 12.570, *P* = 0.001	Antagonistic^f^
Interactive effect of V_exg 100 *μ*M_ × V_end_ × Mg_end_	NS	—

LPO modified by V_exg 200 *μ*M_		
Main effect of V_end_	* F* = 51.672, *P* = 0.000	—
Main effect of Mg_end_	*F* = 16.998, *P* = 0.000	—
Interactive effect of V_end_ × Mg_end_	*F* = 2.999, *P* = 0.088	Antagonistic^b^
Main effect of V_exg 200 *μ*M_	*F* = 372.550, *P* = 0.000	—
Interactive effect of V_exg 200 *μ*M_ × V_end_	*F* = 35.037, *P* = 0.000	Synergistic^e^
Interactive effect of V_exg 200 *μ*M_ × Mg_end_	*F* = 8.299, *P* = 0.005	Antagonistic^f^
Interactive effect of V_exg 200 *μ*M_ × V_end_ × Mg_end_	NS	—

LPO modified by V_exg 400 *μ*M_		
Main effect of V_end_	*F* = 61.594, *P* = 0.000	—
Main effect of Mg_end_	*F* = 14.220, *P* = 0.000	—
Interactive effect of V_end_ × Mg_end_	*F* = 5.271, *P* = 0.025	Antagonistic^b^
Main effect of V_exg 400 *μ*M_	*F* = 1026.907, *P* = 0.000	—
Interactive effect of V_exg 400 *μ*M_ × V_end_	*F* = 42.650, *P* = 0.000	Synergistic^e^
Interactive effect of V_exg 400 *μ*M_ × Mg_end_	*F* = 6.158, *P* = 0.015	Antagonistic^f^
Interactive effect of V_exg 400 *μ*M_ × V_end_ × Mg_end_	NS	—

LPO modified by Mg_exg 100 *μ*M_		
Main effect of V_end_	* F* = 38.869, *P* = 0.000	—
Main effect of Mg_end_	*F* = 49.991, *P* = 0.000	—
Interactive effect of V_end_ × Mg_end_	*F* = 12.331, *P* = 0.001	Antagonistic^b^
Main effect of Mg_exg 100 *μ*M_	NS	—
Interactive effect of Mg_exg 100 *μ*M_ × V_end_	NS	—
Interactive effect of Mg_exg 100 *μ*M_ × Mg_end_	NS	—
Interactive effect of Mg_exg 100 *μ*M_ × V_end_ × Mg_end_	NS	—

LPO modified by Mg_exg 200 *μ*M_		—
Main effect of V_end_	*F* = 39.615, *P* = 0.000	—
Main effect of Mg_end_	*F* = 48.829, *P* = 0.000	—
Interactive effect of V_end_ × Mg_end_	*F* = 16.616, *P* = 0.000	Antagonistic^b^
Main effect of Mg_exg 200 *μ*M_	*F* = 4.838, *P* = 0.031	—
Interactive effect of Mg_exg 200 *μ*M_ × V_end_	NS	—
Interactive effect of Mg_exg 200 *μ*M_ × Mg_end_	NS	—
Interactive effect of Mg_exg 200 *μ*M_ × V_end_ × Mg_end_	NS	—

LPO modified by Mg_exg 400 *μ*M_		
Main effect of V_end_	*F* = 41.824, *P* = 0.000	—
Main effect of Mg_end_	*F* = 53.944, *P* = 0.000	—
Interactive effect of V_end_ × Mg_end_	*F* = 10.024, *P* = 0.002	Antagonistic^b^
Main effect of Mg_exg 400 *μ*M_	*F* = 10.024, *P* = 0.002	—
Interactive effect of Mg_exg 400 *μ*M_ × V_end_	NS	—
Interactive effect of Mg_exg 400 *μ*M_ × Mg_end_	NS	—
Interactive effect of Mg_exg 400 *μ*M_ × V_end_ × Mg_end_	NS	—

V_end_ and Mg_end_: endogenous V (NaVO_3_, SMV) and Mg (MgSO_4_, MS) which were received in combination for 18 weeks; Fe_exg_, V_exg_, Mg_exg_: exogenous Fe (FeSO_4_), V (NaVO_3_) and Mg (MgSO_4_) added to liver supernatants obtained from the SMV-MS-coadministered rats.

^a^Data are presented as *F* values and the levels of significance (*P*). NS: no significant effect.

^b^The effect of V_end_ and Mg_end_ in combination in the presence of Fe_exg 30 *μ*M_ or V_exg 100, 200, 400 *μ*M_ or Mg_exg 100, 200, 400 *μ*M_ < sum of the effects of V_end_ and Mg_end_ alone in the presence of Fe_exg 30 *μM*_ or V_exg 100, 200, 400 *μ*M_ or Mg_exg 100, 200, 400 *μ*M_ (antagonistic interaction).

^c^The effect of V_end_ and Fe_exg 30 *μ*M_  in combination in the presence of Mg_end_ > sum of the effects of V_end_ and Fe_exg 30 *μ*M_  alone in the presence of Mg_end_ (synergistic interaction).

^d^The effect of Mg_end_ and Fe_exg 30 *μ*M_  in combination in the presence of V_end_ < sum of the effects of Mg_end_ and Fe_exg 30 *μ*M_  alone in the presence of V_end_ (antagonistic interaction).

^e^The effect of V_end_ and V_exg 100, 200, 400 *μ*M_  in combination in the presence of Mg_end_ > sum of the effects of V_end_ and V_exg 100, 200, 400 *μ*M_  alone in the presence of Mg_end_ (synergistic interaction).

^f^The effect of Mg_end_ and V_exg 100, 200, 400 *μ*M_  in combination in the presence of V_end_ < sum of the effects of Mg_end_ and V_exg 100, 200, 400 *μ*M_  alone in the presence of V_end_ (antagonistic interaction).

## Abbreviations

Mg_end_: Mg (as MS) administered to rats endogenously
V_end_: V (as SMV) administered to rats endogenously
Mg_exg_: Mg (as MgSO_4_) added exogenously to liver supernatants (LS)
V_exg_: V (as NaVO_3_) added exogenously to liver supernatants (LS)
Fe_exg_: Fe (as FeSO_4_) added exogenously to liver supernatants (LS).
